# Design, Synthesis, and Antimicrobial Activities of 1,2,3-Triazole Glycoside Clickamers

**DOI:** 10.3390/molecules25040790

**Published:** 2020-02-12

**Authors:** Tamer El Malah, Hany F. Nour, Amira A. E. Satti, Bahaa A. Hemdan, Wael A. El-Sayed

**Affiliations:** 1 Photochemistry Department, Chemical Industries Research Division, National Research Centre, 33 El Buhouth Street, P.O. Box 12622 Cairo, Egypt; waelshendy@gmail.com; 2 Chemistry Department, Faculty of Science and Arts in Qurayat, Jouf University, P.O. Box 77425 Qurayat, Saudi Arabia; aalhassan@ju.edu.sa; 3 Chemistry Department, College of Science, Sudan University of Science and Technology, P.O. Box 11116 Khartoum, Sudan; 4 Water Pollution Research Department, Environmental Research Division, National Research Centre, 33 El Buhouth Street, P.O. Box 12622 Cairo, Egypt; bahaa_nrc@yahoo.com; 5 Department of Biosciences and Bioengineering, Indian Institute of Technology Guwahati, P.O. Box 781039 Assam, India; 6 Department of Chemistry, College of Science, Qassim University, P.O. Box 51452 Buraidah, Saudi Arabia

**Keywords:** 1,2,3-triazole, glycoside, click chemistry, antibacterial, antifungal

## Abstract

Bacterial resistance remains a significant threat and a leading cause of death worldwide, despite massive attempts to control infections. In an effort to develop biologically active antibacterial and antifungal agents, six novel aryl-substituted-1,2,3-triazoles linked to carbohydrate units were synthesized through the Cu(I)-catalyzed azide-alkyne cycloaddition CuAAC of substituted-arylazides with a selection of alkyne-functionalized sugars. The chemical structures of the new derivatives were verified using different spectroscopic techniques. The novel clicked 1,2,3-triazoles were evaluated for in vitro antibacterial activity against Gram-positive *Staphylococcus aureus* and Gram-negative *Pseudomonas aeruginosa,* and the obtained results were compared with the activity of the reference antibiotic “Ampicillin”. Likewise, in vitro antifungal activity of the new 1,2,3-triazoles was investigated against *Candida albicans* and *Aspergillus niger* using “Nystatin” as a reference drug. The results of the biological evaluation pointed out that *Staphylococcus aureus* was more susceptible to all of the tested compounds than other examined microbes. In addition, some tested compounds exhibited promising antifungal activity.

## 1. Introduction

**Figure 1:**
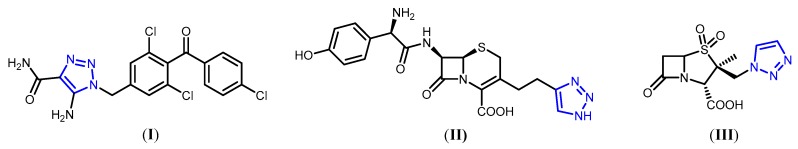
Chemical structures of pharmaceutical drugs containing 1,2,3-triazole rings.

Antimicrobial resistance has emerged as one of the most serious threats to global health. The evolution of bacterial resistance occurs through a wide variety of mechanisms, including acquisition of resistance genes via horizontal gene transfer and mutations [1,2,3,4]. Moreover, overuse and misuse of antibiotics without medical prescription accelerate the emergence and spread of multidrug- resistant bacteria [5,6,7]. Several bacterial strains have evolved sophisticated mechanisms, which allow them to survive and negate the onslaught of antibiotics. For instance, some isolated strains of *Staphylococcus aureus* have developed intrinsic resistance to many kinds of antibiotics, including *β*-lactams, glycopeptides, aminoglycosides, and fluoroquinolones [8,9,10,11]. Rifampicin was used as one of the most effective antibiotics for treatment of *Mycobacterium tuberculosis* through inhibition of the elongation of the messenger RNA. Bacterial resistance to Rifampicin was developed through genetic mutations, which significantly suppress its binding with *β*-subunit of the RNA polymerase [12,13,14]. Some species of *Acinetobacter baumannii* exhibited resistance to currently available antibiotics due to their ability to adapt and develop diverse strategies of resistance, including hydrolysis of *β*-lactam rings of antibiotics, reducing entry of antibiotics into target sites of bacteria, and alteration of bacterial targets by mutations [15,16]. *Pseudomonas aeruginosa* is characterized by its ability to exist in aggregates forming biofilms, which are highly resistant to antibiotics [17]. Therefore, special concern should be devoted to the development of new antimicrobial agents, possessing completely different chemical structures, and working with different modes of actions. The Cu(I)-catalyzed azide-alkyne cycloaddition (CuAAC) reaction offers a versatile synthetic access to the 1,2,3-triazole heterocycle derivatives, which are associated with a wide spectrum of pharmacological activities, including antimycobacterial [18,19,20,21], antitubercular [22,23,24,25], anticancer [26,27,28,29], antiviral [30,31,32,33,34], antidiabetic [35,36,37,38], antifungal [39,40,41,42], anti-HIV [43,44,45,46], anti-inflammatory [47,48,49,50], antimalarial [51,52,53,54], anti- oxidant [55,56,57,58], anti-proliferative [59,60,61,62] properties. The 1,2,3-triazole heterocycle has been utilized as a main component of many pharmaceutical drugs, such as carboxyamidotriazole (CAI) (I), cefatrizine (II), and tazobactum (III) (Figure 1). The synthesis of molecular architectures containing 1,2,3-triazole heterocycles via the CuAAC click approach offers many advantages, such as high stability, yielding, and chemoselectivity of products [63,64]. More importantly, click reactions are insensitive to oxygen or water and can be easily conducted under mild reaction conditions. 1,2,3-Triazoles linked to carbohydrates have attracted widespread attention, on account of their positive effect on enhancing solubility and biological activity [65,66,67,68,69,70,71]. In continuation of our previous work on the synthesis of bioactive molecular architectures based on the 1,2,3-triazole heterocycle [26,27,30], we report in this study the synthesis, characterization, and antimicrobial evaluation of six novel 1,2,3-triazole glycosides using the click chemistry synthetic approach. 

## 2. Results and Discussion

### 2.1. Chemistry

#### Synthesis and Characterization

**Scheme 1:**
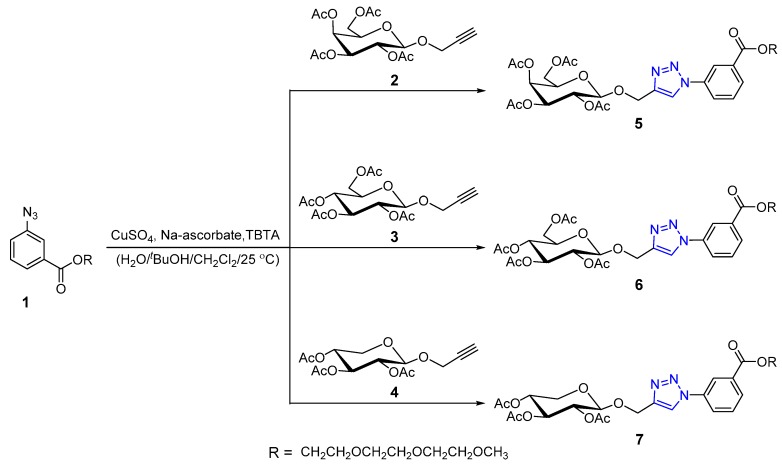
Click synthesis of 1,2,3-triazole glucosides **5**–**7**.

**Scheme 2:**
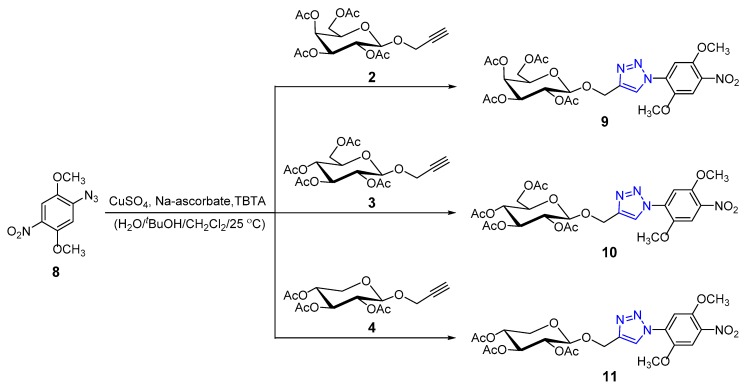
Click synthesis of 1,2,3-triazole glucosides **9–11**.

The Cu(I)-catalyzed cycloaddition of the 2-(2-(2-methoxyethoxy)ethoxy)-ethyl 3-azidobenzoate **1** [26] with the terminal alkyne groups of the acetylated D-galactose **2**, D-glucose **3**, and D-xylose **4** [72] afforded the corresponding 1,2,3-triazole-based glycosides **5**–**7** in good yields (75%–81%) as demonstrated in Scheme 1. The 1,2,3-triazole ring acts as a linker connecting the substituted-aryl and sugar units. Furthermore, it can offer an additional binding motif to biological targets through the basic nitrogen atoms. On the other side, the triethylene glycol units attached to the phenyl ring and the sugar parts provide dual functions, which include binding of biological targets through the electronegative oxygen atoms and enhancing the solubility of products. The chemical structures of the 1,2,3-triazole glucosides **5**–**7** were fully characterized using different spectroscopic techniques (Supporting Information, Figures S1–S6). The ^1^H NMR spectrum of compound **5**, as an example, displayed three signals at *δ* 5.20–5.21, 8.10, and 8.90 ppm, corresponding to the anomeric C*H* of sugar, the single C*H* of triazole ring and the aromatic singlet signals, respectively. This clearly confirms connection of the aryl ring with the sugar part of compound **5** via the heterocyclic 1,2,3-triazole bridge. The ^13^C NMR spectrum of **5** showed the characteristic signals of the carboxylate functional groups attached to the molecule at *δ* 165.5–170.4 ppm, while the anomeric carbon appeared at *δ* 99.3 ppm. The FT-IR spectrum of **5** showed an absorption band at *ν*_max_ 1749 cm^−1^ for the carboxylate C=O groups. The triethylene glycol units incorporated in the structural framework of 1,2,3-triazole glucosides **5**–**7**, along with the acetate units of protected sugars **2**–**4**, are expected to cooperatively contribute in binding of the bacterial targets as similar to the mode of action of *Vancomycin* antibiotic*,* which noncovalently *binds* to the d-Ala–d-Ala section of the bacterial cell wall precursors via hydrogen bonding interactions. In Scheme 2, the click reactions of the substituted aryl azide **8**, which did not incorporate triethylene glycol units, with the alkyne functionalized sugars **2**–**4**, yielded the corresponding 1,2,3-triazole glucosides **9**–**11.**

The 1,2,3-triazole glycosides **9**–**11** were fully characterized using the various spectroscopic tools (Supporting Information, Figures S7–S12). The ^1^H NMR spectrum of the 1,2,3-triazole glycoside **9** showed a multiple signal at *δ* 5.16–5.21 ppm, corresponding to the anomeric C*H* proton of the sugar. It also displayed a characteristic singlet signal at *δ* 8.28 ppm for the 1,2,3-triazole C*H*. Two singlet signals appeared in the aromatic region at *δ* 7.62 and 7.74 ppm, referring to the singlet aromatic C*H* protons.

The appearance of these signals clearly confirms connection of the substituted aryl ring with the sugar unit of compound **9** through the heterocyclic 1,2,3-triazole bridge. The ^13^C NMR spectrum of **9** showed the C=O functional groups at *δ* 169.5–170.4 ppm, while the anomeric carbon appeared at *δ* 100.5 ppm. The FT-IR spectrum displayed the C=O absorption band at *ν*_max_ 1749 cm^−1^.

### 2.2. Antimicrobial Activity

The antimicrobial properties of the novel 1,2,3-triazole glycosides **5**–**7** and **9**–**11** were evaluated towards four different microbial species; namely *S. aureus*, *P. aeruginosa*, *C. albicans,* and *A. niger*. The inhibition zone diameters of the tested compounds are shown in Table 1. The obtained results revealed that the most susceptible organism was *S. aureus*, which was found sensitive to all of the tested compounds.

Generally, compound **5** was the most potent among the series of the newly synthesized glyco- sides and showed distinctive antimicrobial activities. The largest size of the inhibition zone for *S. aureus* was recorded by compound **5** since the size was 21 ± 0.25 and 25 ± 0.15 mm using the disc and well diffusion assay, respectively. On the other side, the results revealed that compound **6** was the one possessing the least antimicrobial activity. Although the lowest inhibition zones were recorded for the two fungal species, it appeared to be moderately and highly sensitive to two of the tested compounds (Table 1). There is a significant zone of inhibition given by compound **5** against *C. albicans* and *A. niger* (15 and 14 mm) using the disc diffusion assay. From the obtained results, it can be concluded that the antimicrobial activities of compound **5** are better than the used reference drug towards *S. aureus*. Meanwhile, interestingly, this compound can eradicate all bacterial and fungal species, while the Ampicillin drug was able to kill bacterial species only and Nystatin drug had only high antifungal activity.

**Figure 2:**
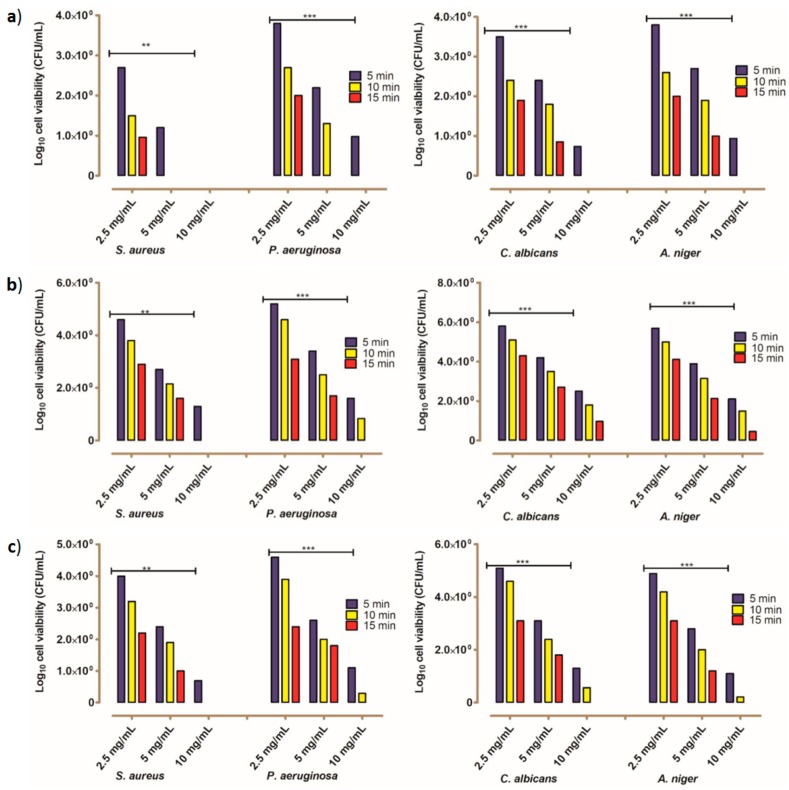
Minimum inhibitory concentration values of the tested compounds (**a**) **5**, (**b**) **6,** and (**c**) **7** towards targeted microbes. The remained viable cell populations at various time intervals of 5, 10, and 15 min, are also shown. Two-way analysis of variance (*ANOVA*) states * indicates low correlation (*p* ≤ 0.05), ** indicates moderate correlation (*p* ≤ 0.01), *** indicates high correlation (*p* ≤ 0.001).

**Figure 3:**
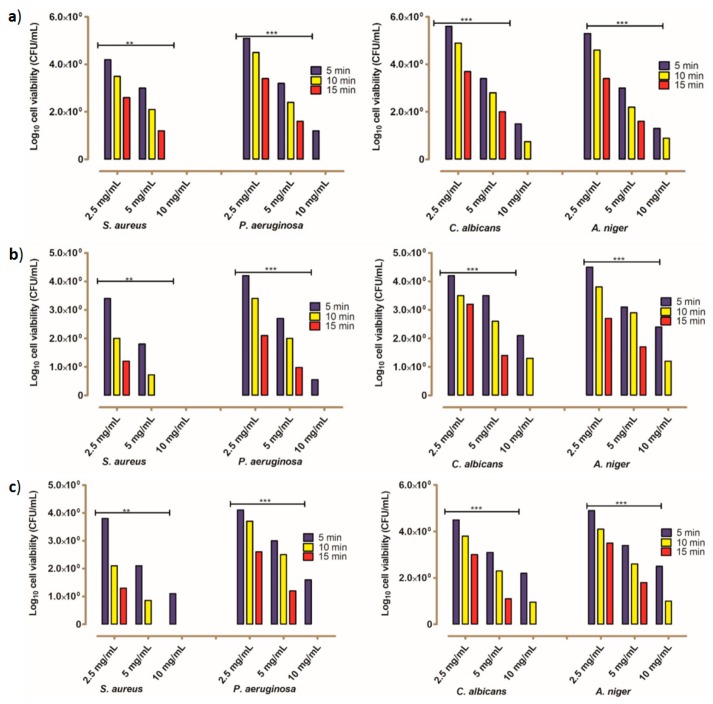
Minimum inhibitory concentration values of the tested compounds (**a**) **9,** (**b**) **10,** and (**c**) **11** towards targeted microbes. The remained viable cell populations at various time intervals of 5, 10, and 15 min, are also shown. Two-way analysis of variance (*ANOVA*) states * indicates low correlation (*p* ≤ 0.05), ** indicates moderate correlation (*p* ≤ 0.01), *** indicates high correlation (*p* ≤ 0.001).

To establish the possible antimicrobial structure-activity relationship, the inactivation effects and the minimum inhibitory concentrations (MICs) values for all of the tested compounds were estimated against the four targeted microbial species. The obtained data through the MIC values revealed variability in the inhibitory concentrations of each compound against the targeted microbial species. From the results of the present study, it was established that the same concentration of several tested compounds could inhibit all microbial growth of all tested microorganisms during different time intervals. The MIC values of compound **5** were found to be 5 mg/mL within 10 for *S. aureus* and 15 min for *P. aeruginosa*, while for fungal species, *C. albicans* and *A. niger*, it was revealed to be 10 mg/mL at 10 min (Figure 2). In the case of compound **6**, 10 mg/mL was capable of killing *S. aureus* and *P. aeruginosa* within 10 and 15 min, but this compound could not inactivate fungal species. Concerning the MIC values of compound **7**, the results figured out the effective concentration of such derivative (10 mg/mL) that could inhibit the growth of *S. aureus* within 10 min and all other tested microbes within 15 min. The results displayed in Figure 3 clearly showed that the value of MIC of compound **9** was 10 mg/mL within 5 min for *S. aureus*, 10 min for *P. aeruginosa*, and 15 min for *C. albicans* and *A. niger.* The MIC values of compounds **10** and **11** against *S. aureus*, *P. aeruginosa*, *C. albicans,* and *A. niger* are 5 mg/mL at 5 min, 10 mg/mL at 10 min, and 10 mg/mL at 15 min (Figure 3). Accordingly, *S. aureus* was more susceptible to all of the tested compounds than other examined microbes, and compound **5** was found to be the most active compound.

In conclusion, the values of MIC using the standard broth dilution technique for all the tested compounds were documented and collected in Table 2. Although no definite structure–activity relationship could be determined based on the applied periods and effective concentrations, there are conclusions concerning structural differences that may influence the antimicrobial activity that can be concluded via correlation with certain characteristic structural features. Accordingly, the six-carbon sugar (galactopyranosyl) in the latter system resulted in higher activities than that containing the xylopyranosyl moiety. However, relatively low differences in activities were revealed by the compounds incorporating the dimethoxynitrophenyl-triazole structure system, which showed that the glycosides with gluco- and xylo- pyranosyl moieties were relatively higher than the triazole glycoside with the D-galactose sugar part. Furthermore, the differences in the observed activities of the glycosides **5** and **6** towards both prokaryotic and eukaryotic organisms revealed the importance of the conformation of the acetyl group at C-4 in the sugar moiety. The latter triazole glycosides have the same aromatic system, but possess the glucopyranosyl and galactopyranosyl epimers which differ only in the conformation of the acetyl group at C-4. Such activity correlation may account for a possible interaction of such group or even may influence the behavior of the sugar part which is also in agreement with the obtained results for similar structural systems [70].

On the other side, the triazole glycoside **5** with the galactopyranosyl moiety showed markedly higher activity than compound **9** which has the same sugar moiety, but both compounds differ in the attached substituted aryl system. This observation showed the effect of aryl substitution on the activity since the glycoside with (tetraoxadodecanoyl) phenyl system was highly active than its analogue dimethoxynitrophenyl-triazole. In addition, from the afforded results, it can be found that the growth inhibitory effects of compounds **10** and **11** were the same against all of the tested microbial strains.

#### 2.2.1. Toxicity Evaluation of the Studied Compounds

The toxicity assay has been performed for safety verification of the designed compounds, and the results showed that all tested compounds were safe and had no hazardous effects for humankind. The readings of EC_50_% were 204%, 320%, 245%, 285%, and 195%, for compounds **5**, **6**, **7**, **9**, **10**, and **11**, respectively. The results also indicated that all EC_50_% values of all tested compounds were more than 100%, which means that such compounds are nontoxic. Moreover, the values EC_50_% of the reference drugs was also lower than 100% (Table 3).

#### 2.2.2. The Kinetic Modeling of the Killing Rate of the Tested Microbes

From the kinetic modeling using pseudo-first-order, the results revealed that the rapidly inhibited strains were the *S. aureus*, while the lowest inhibition was observed for the fungal species. Moreover, the effective concentration of compound **5** was the one that could effectively inhibit the growth of all tested microbial strains within a shorter time than other compounds. It was interesting to note that the tested fungal species were damaged over a longer time (Table 4).

## 3. Experimental

### 3.1. Chemistry

#### 3.1.1. General Procedures

All the reagents used for the reactions were purchased from Alfa Aesar Germany or Fisher (UK) and were used as obtained without further purification. The reactions were monitored by thin layer chromatography (TLC). TLC was performed on Macherey-Nagel aluminium-backed plates, pre-coated with silica gel 60 (UV_254_). Column chromatography was carried out on silica gel 60 (0.040–0.063 mm) under flash conditions. The ^1^H NMR and ^13^C NMR spectra were measured on a JEOL 400 MHz for ^1^H NMR and 100 MHz for ^13^C NMR in CDCl_3_, Microanalytical Center, Faculty of Pharmacy, Cairo University, Egypt. The chemical shifts (*δ*) were expressed in ppm relative to the standard TMS as an internal reference. The coupling constants (*J*) were given in Hz. The microanalytical data were carried out on a Vario El-Mentar instrument, Microanalytical Center, Cairo University, Egypt. The melting points were determined in open glass capillaries using an Electrothermal IA 9000 SERIES digital melting point apparatus (Electrothermal, UK) and are uncorrected. The FT-IR spectra were recorded on a Shimadzu IR 8400s spectrophotometer (KBr, *ν*_max_/cm^−1^) at the Micro Analytical Laboratory, Cairo University, Egypt. The aromatic azides (**1** and **8**) and acetylenic sugars (**2**, **3,** and **4**) were prepared according to the reported procedures in the literature [26,27,72].

#### 3.1.2. General Click Chemistry Procedure

A three-necked flask was charged with alkynes compounds (**2**, **3**, or **4**) (1 equivalent), and aryl azides (**1** or **8**) (1 equivalent), sodium ascorbate (0.3 equivalent), TBTA (0.15 equivalent), and a solvent mixture of H_2_O/*^t^*BuOH/CH_2_Cl_2_ (1/2/8, 60 mL). The flask was evacuated and flushed with argon repeatedly (three cycles). CuSO_4_.5H_2_O was added (0.15 equivalent) and the mixture was stirred for two days at room temperature in the dark. After consumption of alkynes (**2**, **3**, or **4**) as indicated by TLC monitoring, the mixture was diluted with CH_2_Cl_2_ and transferred into a separating funnel. The organic phase was washed with an aqueous ethylenediaminetetraacetic acid (EDTA) disodium salt (EDTA-Na_2_) solution (3×), the aqueous phase was extracted with CH_2_Cl_2_ (3×), and aqueous saturated NaCl solution (1×) (60 mL volume). After drying over MgSO_4_, filtration, and removal of the solvent under vacuum the target compounds were obtained after purification by column chromatography.

#### 3.1.3. *(2R,3R,4S,5S,6R)-2-((1-(3-(2,5,8,11-tetraoxadodecanoyl)phenyl)-1H-1,2,3-triazol-4-yl)methoxy)-6-(a-cetoxymethyl)tetrahydro-2H-pyran-3,4,5-triyl triacetate (5)*

The title compound was separated and purified by column chromatography (Petroleum ether/ethyl acetate 8/2) as a brown solid (81%). Mp 155–156 °C. TLC (Petroleum ether/ethyl acetate 8/2) R*_f_* = 0.28. FT-IR (KBr, *ν*_max_/cm^−1^): 1749 (C=O). ^1^H NMR (400 MHz, CDCl_3_): *δ* (ppm) = 1.91, 1.94 (ss, 6 H, 2 COC*H*_3_), 2.03 (s, 3 H, COC*H*_3_), 2.13 (s, 3 H, COC*H*_3_), 3.37 (s, 3 H, OC*H*_3_), 3.62 (s, 3 H, C*H*_2_), 4.09–4.10 (m, 6 H, 3 C*H*_2_), 4.25–4.27 (ss, 3 H, C*H*+C*H*_2_), 4.76 (t, ^3^*J* = 8.51 Hz, 2 H, C*H*_2_), 4.87–5.19 (m, 6 H, 2 C*H*+2C*H*_2_), 5.20, 5.21 (m, 1 H, C*H*), 5.28 (s, 1 H, C*H*), 7.56 (s, 1 H, Ar*H*), 7.69 (s, 1 H, Ar*H*), 8.10 (s, 1 H, Ar*H*_triazole_), 8.41, 8.45 (ss, 1 H, Ar*H*), 8.90 (s, 1 H, Ar*H*). ^13^C NMR (75 MHz, CDCl_3_): *δ* (ppm) = 20.7 (COCH_3_), 20.8 (COCH_3_), 20.9 (COCH_3_), 53.1 (OCH_3_), 61.7 (CH_2_), 62.9 (CH_2_), 67.8 (CH_2_), 67.8 (CH_2_), 69.0 (CH), 70.4 (CH_2_), 70.6 (CH_2_), 71.2 (CH_2_), 71.7 (CH), 72.2 (CH_2_), 72.4 (CH), 73.4 (CH), 99.3 (CHOC_suger_), 123.4 (C_Ar_), 124.6 (C_Ar_), 128.1 (CH_triazole_), 128.5 (C_Ar_), 129.2 (C_Ar_), 136.7 (C_Ar_), 140.5 (C_Ar_), 144.1 (C_triazole_CH_2_O), 165.5, 169.5, 169.9, 170.4, 170.4 (C=O). Elemental analysis, calc. (%) for C_31_H_41_N_3_O_15_: *C* 53.52, *H* 5.94, *N* 6.04; found: *C* 53.74, *H* 6.17, *N* 5.86.

#### 3.1.4. *(2R,3R,4S,5R,6R)-2-((1-(3-(2,5,8,11-tetraoxadodecanoyl)phenyl)-1H-1,2,3-triazol-4-yl)methoxy)-6-(a-cetoxymethyl)tetrahydro-2H-pyran-3,4,5-triyl triacetate (6)*

The title compound was separated and purified by column chromatography (Petroleum ether/ethyl acetate 8/2) as a yellow oil (78%). TLC (Petroleum ether/ethyl acetate 8/2) R*_f_* = 0.30. FT-IR (KBr, *ν*_max_/cm^−1^): 1751 (C=O). ^1^H NMR (400 MHz, CDCl_3_): *δ* (ppm) = 1.93, 1.94 (ss, 6 H, 2 COC*H*_3_), 1.96 (s, 3 H, COC*H*_3_), 2.01 (s, 3 H, COC*H*_3_), 3.28 (s, 3 H, OC*H*_3_), 3.45-3.47 (m, 2 H, C*H*_2_), 3.56–3.73 (m, 6 H, 3 C*H*_2_), 3.79 (t, ^3^*J* = 4.76 Hz, 2 H, C*H*_2_), 4.09–4.13, 2 H, OC*H*_2_), 4.68, 4.70 (ss, 2 H, OC*H*_2_), 4.85, 4.88 (ss, 1 H, C*H*), 4.95–5.06 (m, 3 H, C*H*), 5.16 (m, 1 H, C*H*), 7.57 (t, ^3^*J* = 7.95 Hz, 1 H, Ar*H*), 7.94 (d, ^2^*J* = 9.21 Hz, 1 H, Ar*H*), 8.05 (s, 1 H, Ar*H*_triazole_), 8.07 (d, ^2^*J* = 7.83 Hz, 1 H, Ar*H*), 8.31 (s, 1 H, Ar*H*). ^13^C NMR (75 MHz, CDCl_3_): *δ* (ppm) = 20.5 (COCH_3_), 20.6 (COCH_3_), 20.7 (COCH_3_), 58.9 (OCH_3_), 61.8 (CH_2_), 62.7 (CH_2_), 64.6 (CH_2_), 68.3 (CH_2_), 69.0 (CH), 70.5 (CH_2_), 70.6 (CH_2_), 70.6 (CH_2_), 71.2 (CH), 71.8 (CH_2_), 72.0 (CH), 72.7 (CH), 100.0 (CHOC_suger_), 121.2 (C_Ar_), 121.3 (C_Ar_), 124.8 (CH_triazole_), 130.0 (C_Ar_), 130.0 (C_Ar_), 131.9 (C_Ar_), 137.0 (C_Ar_), 145.0 (C_triazole_CH_2_O), 165.1, 169.4, 169.4, 170.1, 170.6 (C=O). Elemental analysis, calc. (%) for C_31_H_41_N_3_O_15_: *C* 53.52, *H* 5.94, *N* 6.04; found: *C* 53.79, *H* 5.76, *N* 5.88.

#### 3.1.5. *(2R,3R,4S,5R)-2-((1-(3-(2,5,8,11-tetraoxadodecanoyl)phenyl)-1H-1,2,3-triazol-4-yl)methoxy)tetrahyd-ro-2H-pyran-3,4,5-triyl triacetate (7)*

The title compound was separated and purified by column chromatography (Petroleum ether/ethyl acetate 8/2) as a yellow oil (75%). TLC (Petroleum ether/ethyl acetate 8/2) R*_f_* = 0.32. FT-IR (KBr, *ν*_max_/cm^−1^): 1749 (C=O). ^1^H NMR (400 MHz, CDCl_3_): *δ* (ppm) = 1.96 (s, 6 H, 2COC*H*_3_), 1.98 (s, 3 H, COC*H*_3_), 3.28 (s, 3 H, OC*H*_3_), 3.35–3.40 (m, 2 H, C*H*_2_), 3.44–3.47 (m, 2 H, C*H*_2_), 3.56–3.66 (m, 6 H, 3 C*H*_2_), 3.78–3.80 (m, 1 H, C*H_a_*), 4.08–4.12 (m, 1 H, C*H_b_*), 4.44–4.47 (m, 2 H, OC*H*_2_), 4.63, 4.65 (ss, 2 H, OC*H*_2_), 4.80–4.96 (m, 3 H, 2 C*H*), 5.12 (m, 1 H, C*H*), 7.56 (t, ^3^*J* = 5.28 Hz, 1 H, Ar*H*), 7.96 (d, ^2^*J* = 8.10 Hz, 1 H, Ar*H*), 8.03 (s, 1 H, Ar*H*_triazole_), 8.06 (d, ^2^*J* = 7.77 Hz, 1 H, Ar*H*), 8.30 (s, 1 H, Ar*H*). ^13^C NMR (75 MHz, CDCl_3_): *δ* (ppm) = 20.6 (COCH_3_), 20.7 (COCH_3_), 58.9 (OCH_3_), 62.1 (CH_2_), 62.2 (CH_2_), 64.6 (CH_2_), 68.7 (CH), 69.0 (CH_2_), 70.5 (CH), 70.5 (CH_2_), 70.6 (CH), 70.7 (CH_2_), 71.3 (CH_2_), 71.8 (CH_2_), 99.9 (CH_2_OC_suger_), 121.1 (C_Ar_), 121.2 (C_Ar_), 124.8 (CH_triazole_), 129.9 (C_Ar_), 130.0 (C_Ar_), 131.9 (C_Ar_), 137.0 (C_Ar_), 145.1 (C_triazole_CH_2_O), 169.1, 169.4, 169.8, 169.9 (C=O). Elemental analysis, calc. (%) for C_28_H_37_N_3_O_13_: *C* 53.93, *H* 5.98, *N* 6.74; found: *C* 53.68, *H* 6.21, *N* 6.57.

#### 3.1.6. *(2R,3S,4S,5R,6R)-2-(acetoxymethyl)-6-((1-(2,5-dimethoxy-4-nitrophenyl)-1H-1,2,3-triazol-4-yl)meth-oxy)tetrahydro-2H-pyran-3,4,5-triyl triacetate (9)*

The title compound was separated and purified by column chromatography (Petroleum ether/ethyl acetate 8/2) as a brown solid (77%). Mp 118–119 °C. TLC (Petroleum ether/ethyl acetate 8/2) R*_f_* = 0.29. FT-IR (KBr, *ν*_max_/cm^−1^): 1749 (C=O). ^1^H NMR (400 MHz, CDCl_3_): *δ* (ppm) = 1.93 (s, 3 H, COC*H*_3_), 1.98, 199 (ss, 6 H, 2 COC*H*_3_), 2.09 (s, 3 H, COC*H*_3_), 3.62–3.66 (m, 1 H, C*H*), 3.92, 3.95 (ss, 6 H, 2 OC*H*_3_), 4.03–4.16 (m, 2 H, C*H_a_*_+*b*_), 4.64,4.66 (ss, 1 H, C*H*), 4.81, 4.84 (ss, 2 H, 2 C*H*), 4.96–5.02 (m, 2 H, C*H*_2_), 5.16–5.21 (m, 1 H, C*H*), 7.62 (s, 1 H, Ar*H*), 7.74 (s, 1 H, Ar*H*), 8.28 (s, 1 H, Ar*H*_triazole_). ^13^C NMR (75 MHz, CDCl_3_): *δ* (ppm) = 20.5 (COCH_3_), 20.6 (COCH_3_), 20.7 (COCH_3_), 20.7 (COCH_3_), 56.9 (OCH_3_), 57.3 (OCH_3_), 61.2 (CH_2_), 62.7 (CH_2_), 67.0 (CH), 68.8 (CH), 70.7 (CH), 70.8 (CH), 100.5 (CH_2_OC_suger_), 110.1 (C_Ar_), 110.4 (C_Ar_), 125.0 (CH_triazole_), 130.1 (C_Ar_), 138.2 (C_Ar_NO_2_), 143.1 (C_triazole_CH_2_O), 144.4 (C_Ar_OCH_3_), 147.9 (C_Ar_OCH_3_), 169.5, 170.1, 170.2, 170. 4 (C=O). Elemental analysis, calc. (%) for C_25_H_30_N_4_O_14_: *C* 49.18, *H* 4.95, *N* 9.18; found: *C* 49.34, *H* 5.16, *N* 9.37.

#### 3.1.7. *(2R,3R,4S,5R,6R)-2-(acetoxymethyl)-6-((1-(2,5-dimethoxy-4-nitrophenyl)-1H-1,2,3-triazol-4-yl)meth-oxy)tetrahydro-2H-pyran-3,4,5-triyl triacetate (10)*

The title compound was separated and purified by column chromatography (Petroleum ether/ethyl acetate 8/2) as a yellow solid (80%). Mp 145–146 °C. TLC (Petroleum ether/ethyl acetate 8/2) R*_f_* = 0.33. FT-IR (KBr, *ν*_max_/cm^−1^): 1750 (C=O). ^1^H NMR (400 MHz, CDCl_3_): *δ* (ppm) = 1.93 (ss, 6 H, 2 COC*H*_3_), 1.96 (s, 3 H, COC*H*_3_), 2.02 (s, 3 H, COC*H*_3_), 3.92-3.95 (m, 1 H, C*H*), 4.08, 4.11 (ss, 6 H, 2 OC*H*_3_), 4.20–4.21 (m, 1 H, C*H_a_*), 4.21–4.23 (m, 1 H, C*H_b_*), 4.67, 4.69 (ss, 1 H, C*H*), 4.82, 4.85 (ss, 1 H, C*H*), 4.95–5.06 (m, 3 H, C*H*_2_+C*H*), 5.16 (m, 1 H, C*H*), 7.62 (s, 1 H, Ar*H*), 7.74 (s, 1 H, Ar*H*), 8.28 (s, 1 H, Ar*H*_triazole_). ^13^C NMR (75 MHz, CDCl_3_): *δ* (ppm) = 20.5 (COCH_3_), 20.6 (COCH_3_), 20.7 (COCH_3_), 20.7 (COCH_3_), 56.9 (OCH_3_), 57.3 (OCH_3_), 61.8 (CH_2_), 62.6 (CH_2_), 68.3 (CH), 71.2 (CH), 71.9 (CH), 72.6 (CH), 99.9 (CH_2_OC_suger_), 110.2 (C_Ar_), 110.3 (C_Ar_), 125.1 (CH_triazole_), 130.0 (C_Ar_), 138.2 (C_Ar_NO_2_), 143.1 (C_triazole_CH_2_O), 144.2 (C_Ar_OCH_3_), 147.8 (C_Ar_OCH_3_), 169.3, 169.4, 170.1, 170. 6 (C=O). Elemental analysis, calc. (%) for C_25_H_30_N_4_O_14_: *C* 49.18, *H* 4.95, *N* 9.18; found: *C* 48.95, *H* 5.14, *N* 9.06.

#### 3.1.8. *(2R,3R,4S,5R)-2-((1-(2,5-dimethoxy-4-nitrophenyl)-1H-1,2,3-triazol-4-yl)methoxy)tetrahydro-2H-py-ran-3,4,5-triyl triacetate (11)*

The title compound was separated and purified by column chromatography (Petroleum ether/ethyl acetate 8/2) as a yellow solid (83%). Mp 142–143 °C. TLC (Petroleum ether/ethyl acetate 8/2) R*_f_* = 0.34. FT-IR (KBr, *ν*_max_/cm^−1^): 1751 (C=O). ^1^H NMR (400 MHz, CDCl_3_): *δ* (ppm) = 2.00, 201 (ss, 6 H, 2 COC*H*_3_), 2.04 (s, 3 H, COC*H*_3_), 3.40–3.45 (m, 1 H, C*H_a_*), 3.98, 4.00 (ss, 6 H, 2 OC*H_3_*), 4.15–4.17 (m, 1 H, C*H_b_*), 4.68, 4.69 (ss, 1 H, C*H*), 4.84, 4.87 (ss, 1 H, C*H*), 5.01–4.93 (m, 3 H, C*H*_2_+C*H*), 5.16 (m, 1 H, C*H*), 7.67 (s, 1 H, Ar*H*), 7.79 (s, 1 H, Ar*H*), 8.33 (s, 1 H, Ar*H*_triazole_). ^13^C NMR (75 MHz, CDCl_3_): *δ* (ppm) = 20.6 (COCH_3_), 20.7 (COCH_3_), 56.9 (OCH_3_), 57.3 (OCH_3_), 62.1 (CH_2_), 62.2 (CH_2_), 68.8 (CH), 70.7 (CH), 71.2 (CH), 99.9 (CH_2_OC_suger_), 110.1 (C_Ar_), 110.3 (C_Ar_), 125.0 (CH_triazole_), 130.0 (C_Ar_), 138.1 (C_Ar_NO_2_), 143.1 (C_triazole_CH_2_O), 144.3 (C_Ar_OCH_3_), 147.8 (C_Ar_OCH_3_), 169.4, 169.8, 170.0 (C=O). Elemental analysis, calc. (%) for C_22_H_26_N_4_O_12_: *C* 49. 07, *H* 4.87, *N* 10.41; found: *C* 49.32, *H* 5.13, *N* 10.59.

### 3.2. Biology

#### 3.2.1. Preparation of Stock Solutions for Each Studied Designed Compound

Two hundred microliters of each of tested compound were completely solubilized in 300 µL of 10% dimethyl sulphoxide, and partly dissolved compounds were agitated under constant condition (150 RPM for 3 h at 35 °C) to a final concentration of 50 mg/mL. All dissolved compounds were kept in labeled and tightly closed sterile falcon tube.

#### 3.2.2. Bacterial Cultures and Growth Conditions

A stock of four targeted problematic microbes, including Gram-negative species (*Pseudomonas aeruginosa* ATCC 10145), Gram-positive species (*Staphylococcus aureus* ATCC ATCC 43300), yeast (*Candida albicans* ATCC 10231), and mold (*Aspergillus niger* ATCC 6275), which was maintained at −20 °C, was initially proliferated and prepared according to the literature [73]. The numbers of targeted microbial cells were almost 10^6^ CFU/mL. All experimental trials were repeated three times during different time spans.

#### 3.2.3. Antimicrobial Activity Assay

Antimicrobial susceptibility testing of six tested compounds (**5**, **6**, **7**, **9**, **10**, and **11**) and solvent was performed using both disc and well diffusion agar methods [74]. Concisely, a 100 µL of each microbial culture was carefully distributed onto the surface of Mueller-Hinton agar (MHA) plates using a sterile cotton swab. For disc-diffusion assay, saturated discs with each dissolved compound and one for DMSO solvent were placed on the surface of a MTA medium. While in case of the well diffusion assay, seven wells with 8 mm diameter were drilled into the bottom of a MHA medium under aseptic environments and filled with 100 μL of each tested compound and solvent. After that, all inoculated MHA plates were incubated overnight at 37 °C for bacterial species and 30 °C for fungal species. Distilled water served as negative controls while standard antibiotic discs of Ampicillin and Nystatin were used as the positive controls. The thicknesses of the clear zones around the discs and wells were estimated in mm after the optimum incubation period (24 h). The obtained results were recorded as the mean ± standard deviation.

#### 3.2.4. Minimum Inhibitory Concentration (MIC)

The broth dilution assay was applied for determining the MIC values for each tested compound. From a stock solution of each tested compound (50 mg/mL), three different concentrations (2.5, 5, and 10 mg/mL) of each compound were transferred to a tube containing 20 µL of fresh pathogen microbial culture to estimate the lethal concentrations. After three different time intervals (5, 10, 15, and 20 min), the densities of surviving cells were counted using the plate count viability assay [75].

#### 3.2.5. Estimation of Decay Rate Using Pseudo-First-Order Kinetic Modeling

Pseudo-first-order kinetic modeling was employed to calculate the decay rate (*K*_1_) for each target pathogen, establishing the number of microbial cells that were treated with a low concentration of each compound at different contact times (Nt) with respect to the original counts (N0) according to Equation (1) [76]:

(1) $$\log\left(q_{e}-q_{t}\right)=\log q_{e}-\frac{K_{1}t}{2.303}$$

where *K*_1_ (1 min^−1^) is the rate of inactivation, *qe* (mg g^−1^), and *q_t_* (mg g^−1^) are the amounts sorbed at equilibrium and at time *t* (min), respectively. A straight line of ln (*q_e_* − *q_t_*) versus *t* suggests that this kinetic model applies to the data. For obtaining the relationship between the rate of decayed cells and exposure time, a linear regression (R^2^) was fulfilled.

#### 3.2.6. Toxicity Valuation of the Tested Compounds

The toxicity proportion was measured using the Microtox^®^ Model 500 (M500) analyzer (Modern Water Inc, New Castle, DE, USA) and cytotoxicity assay to exploit these compounds without hazardous effects on individuals or other living creatures in different biomedical applications [77].

#### 3.2.7. Statistical Analyses

Statistical analysis was performed using the software version 5.0 (USA) of GraphPad Prism. Two-way analysis of variance (*ANOVA*) and Student’s *t*-test were accomplished to evaluate significance (*p* < 0.05) between the concentrations of tested compounds and log counts of viable cells of target microbes.

## 4. Conclusions

In summary, six novel 1,2,3-triazole glucosides were synthesized using the standard-click reactions of aryl azides **1** and **8** with sugars **2**–**4**. The antibacterial activity of the new triazoles was evaluated against *S. aureus* and *P. aeruginosa* in comparison with “Ampicillin”, while the antifungal activity was assessed against *C. albicans* and *A. niger* relative to the reference drug “Nystatin”. The antimicrobial evaluation of the new 1,2,3-triazole glucosides revealed a potent activity of compound **5** in comparison with other tested compounds, which obviously highlighted the positive impact of the triethylene glycol sidearm and the acetylated sugar unit on the enhanced biological activity.

## Figures and Tables

**Table 1:** The diameters of the inhibition zone of the tested compounds around tested pathogenic microbes.

Tested Compounds	Diffusion Assays	Inhibition Zone Diameters (mm)			
		*S. aureus*	*P. aeruginosa*	*C. albicans*	*A. niger*
**5**	Disc	21 ± 0.25	17 ± 0.21	15 ± 0.22	14 ± 0.25
	Well	25 ± 0.15	19 ± 0.32	17 ± 0.15	16 ± 0.18
**6**	Disc	10 ± 0.23	8 ± 0.25	7 ± 0.23	6 ± 0.15
	Well	12 ± 0.21	10 ± 0.27	9 ± 0.17	8 ± 0.24
**7**	Disc	13 ± 0.11	10 ± 0.21	8 ± 0.26	9 ± 0.22
	Well	16 ± 0.23	13 ± 0.24	10 ± 0.18	11 ± 0.27
**9**	Disc	14 ± 0.28	10 ± 0.15	8 ± 0.18	9 ± 0.24
	Well	16 ± 0.19	12 ± 0.31	10 ± 0.26	10 ± 0.29
**10**	Disc	16 ± 0.28	12 ± 0.15	11 ± 0.18	10 ± 0.24
	Well	19 ± 0.21	16 ± 0.31	13 ± 0.26	12 ± 0.29
**11**	Disc	18 ± 0.23	15 ± 0.11	13 ± 0.31	11 ± 0.17
	Well	21 ± 0.18	18 ± 0.27	16 ± 0.28	14 ± 0.21
Ampicillin	Disc	22 ± 0.23	18 ± 0.23	0	0
	Well	24 ± 0.19	20 ± 0.19	0	0
Nystatin	Disc	0	0	19 ± 0.15	14 ± 0.21
	Well	0	0	22 ± 0.20	16 ± 0.23

Data values are presented as mean + SD (*n* = 3).

**Table 2:** Summaries of the estimated values of minimum inhibitory concentration (MIC) for the tested compounds towards targeted microbes.

Studied Designed Compounds	Targeted Microbes	Estimated Values of MIC in mg/mL	
		Concentration (mg/mL)	Contact Time (min)
**5**	*S. aureus*	5	10
	*P. aeruginosa*	5	15
	*C. albicans*	10	10
	*A. niger*	10	10
**6**	*S. aureus*	10	10
	*P. aeruginosa*	10	15
	*C. albicans*	10	>15
	*A. niger*	10	>15
**7**	*S. aureus*	10	10
	*P. aeruginosa*	10	15
	*C. albicans*	10	15
	*A. niger*	10	15
**9**	*S. aureus*	10	5
	*P. aeruginosa*	10	10
	*C. albicans*	10	15
	*A. niger*	10	15
**10**	*S. aureus*	5	15
	*P. aeruginosa*	10	10
	*C. albicans*	10	15
	*A. niger*	10	15
**11**	*S. aureus*	5	15
	*P. aeruginosa*	10	10
	*C. albicans*	10	15
	*A. niger*	10	15
**Ampicillin**	*S. aureus*	2.5	10
	*P. aeruginosa*	5	10
	*C. albicans*	>10	>15
	*A. niger*	>10	>15
**Nystatin**	*S. aureus*	>10	>15
	*P. aeruginosa*	>10	>15
	*C. albicans*	2.5	15
	*A. niger*	5	15

**Table 3:** The values of Microtox^®^ EC_50_ percent concentrations of all studied designed compounds.

Studied Compounds	EC_50_% Concentrations	EC_50_% Degree	Toxicity Level
**5**	204	0–19	Extremely toxic
**6**	320	20–39	Very toxic
**7**	245	40–59	Toxic
**9**	285	60–79	Moderately toxic
**10**	195	≥100	Nontoxic and safe
**11**	232		
Ampicillin	292		
Nystatin	254		

**Table 4:** The killing rate of targeted microbes by 2.5 mg/mL of the designed compounds using Pseudo-first-order kinetic modeling.

Studied Designed Compounds	Targeted Microbes	Kinetic Modeling	
		*K*_1_ (min^−1^)	*R* ^2^
**5**	*S. aureus*	0.0918	0.9983
	*P. aeruginosa*	0.0743	0.9912
	*C. albicans*	0.0657	0.9818
	*A. niger*	0.0613	0.9824
**6**	*S. aureus*	0.0412	0.9947
	*P. aeruginosa*	0.0215	0.9915
	*C. albicans*	0.0201	0.9832
	*A. niger*	0.0198	0.9872
**7**	*S. aureus*	0.0515	0.9963
	*P. aeruginosa*	0.0269	0.9950
	*C. albicans*	0.0315	0.9867
	*A. niger*	0.0342	0.9836
**9**	*S. aureus*	0.0558	0.9737
	*P. aeruginosa*	0.0247	0.9931
	*C. albicans*	0.0379	0.9812
	*A. niger*	0.0354	0.9841
**10**	*S. aureus*	0.0816	0.9912
	*P. aeruginosa*	0.0686	0.9894
	*C. albicans*	0.0548	0.9881
	*A. niger*	0.0526	0.9862
**11**	*S. aureus*	0.0845	0.9986
	*P. aeruginosa*	0.0676	0.9927
	*C. albicans*	0.0593	0.9880
	*A. niger*	0.0517	0.9832

## Supplementary Materials

The following are available online, Figure S1: ^1^H NMR spectrum of 1,2,3-triazole glucoside 5 (CDCl3, 400 MHz), Figure S2: ^13^C NMR spectrum of 1,2,3-triazole glucoside 5 (CDCl3, 100 MHz), Figure S3: ^1^H NMR spectrum of 1,2,3-triazole glucoside 6 (CDCl3, 400 MHz); Figure S4: ^13^C NMR spectrum of 1,2,3-triazole glucoside 6 (CDCl3, 100 MHz), Figure S5: ^1^H NMR spectrum of 1,2,3-triazole glucoside 7 (CDCl3, 400 MHz), Figure S6: ^13^C NMR spectrum of 1,2,3-triazole glucoside 7 (CDCl3, 100 MHz), Figure S7: ^1^H NMR spectrum of 1,2,3-triazole glucoside 9 (CDCl3, 400 MHz), Figure S8: ^13^C NMR spectrum of 1,2,3-triazole glucoside 9 (CDCl3, 100 MHz), Figure S9: ^1^H NMR spectrum of 1,2,3-triazole glucoside 10 (CDCl3, 400 MHz), Figure S10: ^13^C NMR spectrum of 1,2,3-triazole glucoside 10 (CDCl3, 100 MHz), Figure S11: ^1^H NMR spectrum of 1,2,3-triazole glucoside 11 (CDCl3, 400 MHz), Figure S12: ^13^C NMR spectrum of 1,2,3-triazole glucoside 11 (CDCl3, 100 MHz).
