# Supplementary material for: Design, Synthesis, and Antimicrobial Activities of 1,2,3-Triazole Glycoside Clickamers
Source: Molecules. 2020 Feb 12;25(4):790. doi: 10.3390/molecules25040790 (PMC7071105; doi:10.3390/molecules25040790)
Supplement: Supplementary file 1 [file molecules-25-00790-s001.pdf]

Design, Synthesis and Antimicrobial Activities of 1,2,3-Triazole Glycoside Clickamers

Tamer El Malah[1,*], Hany F. Nour[1,*], Amira A. E. Satti[2,3], Bahaa A. Hemdan[4,5] and Wael A. El-Sayed[1,6]

[1] Photochemistry Department, Chemical Industries Research Division, National Research Centre, 33 El Buhouth Street, P.O. Box 12622 Cairo, Egypt; tmara_nrc3000@yahoo.com; hany.nour@daad-alumni.de
[2] Chemistry Department, Faculty of Science and Arts in Qurayat, Jouf University, P.O. Box 77425, Kingdom of Saudi Arabia; aalhassan@ju.edu.sa
[3] Chemistry Department, College of Science, Sudan University of Science and Technology, P.O. Box 11116 Khartoum, Sudan
[4] Water Pollution Research Department, Environmental Research Division, National Research Centre, 33 El Buhouth Street, P.O. Box 12622 Cairo, Egypt; bahaa_nrc@yahoo.com
[5] Department of Biosciences and Bioengineering, Indian Institute of Technology Guwahati, P.O. Box 781039 Assam, India
[6] Department of Chemistry, College of Science, Qassim University, P.O. Box 51452 Buraidah, Kingdom of Saudi Arabia; waelshendy@gmail.com
* Correspondence: tmara_nrc3000@yahoo.com (T.E.M.); hany.nour@daad-alumni.de (H.F.N.)

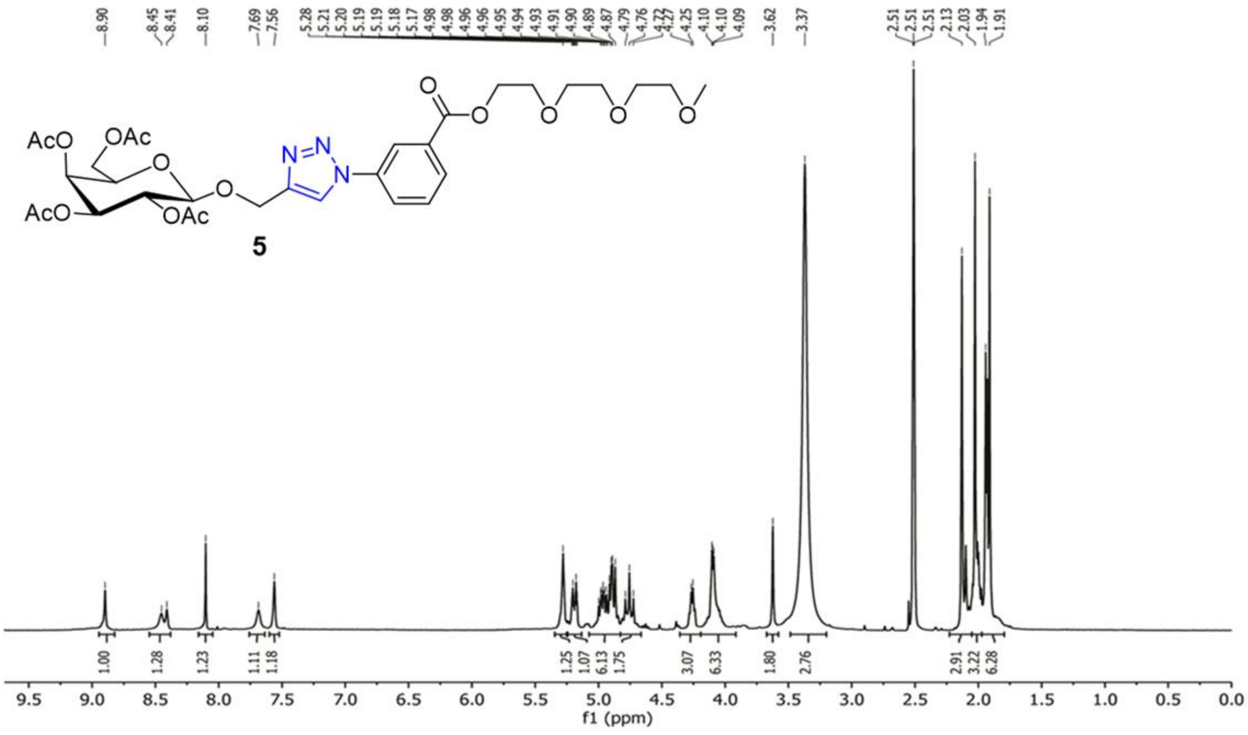

Figure 1. ^{1}H NMR spectrum of 1,2,3-triazole glucoside **5** ($CDCl_3$, 400 MHz).

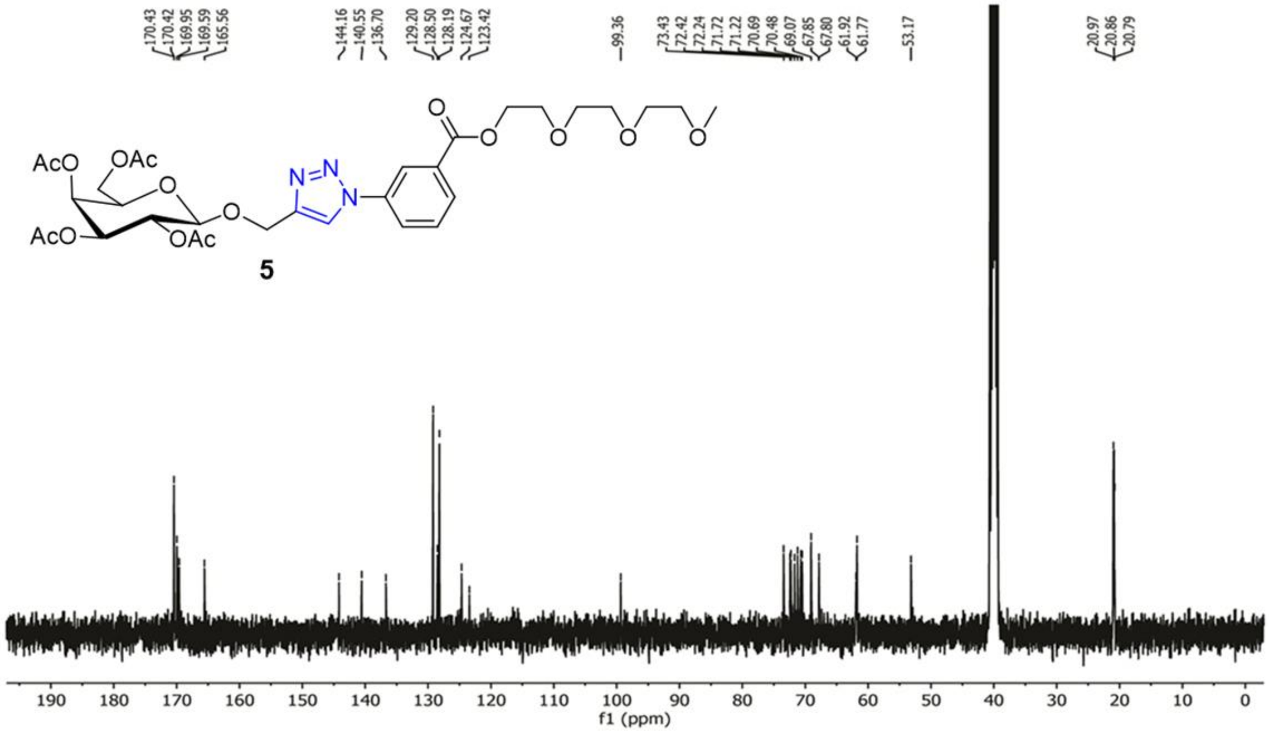

Figure 2. ^{13}C NMR spectrum of 1,2,3-triazole glucoside **5** ($CDCl_3$, 100 MHz).

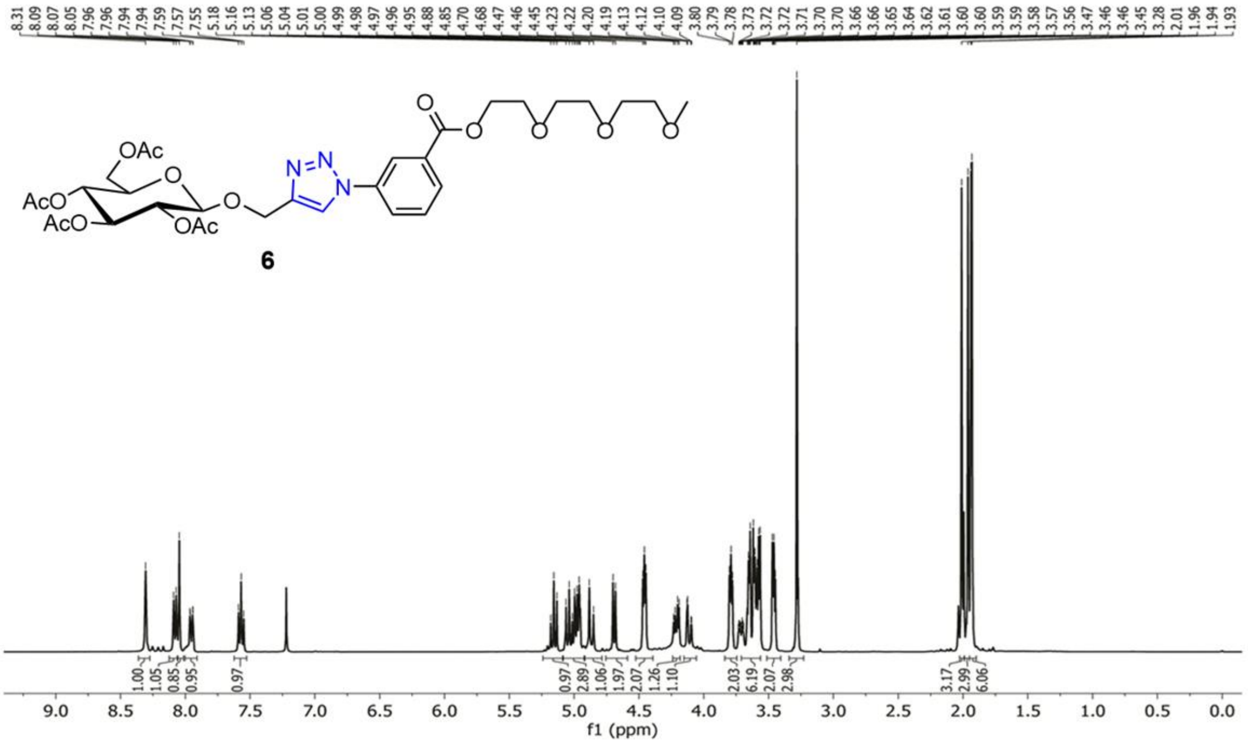

Figure 3. ^{1}H NMR spectrum of 1,2,3-triazole glucoside **6** ($CDCl_3$, 400 MHz).

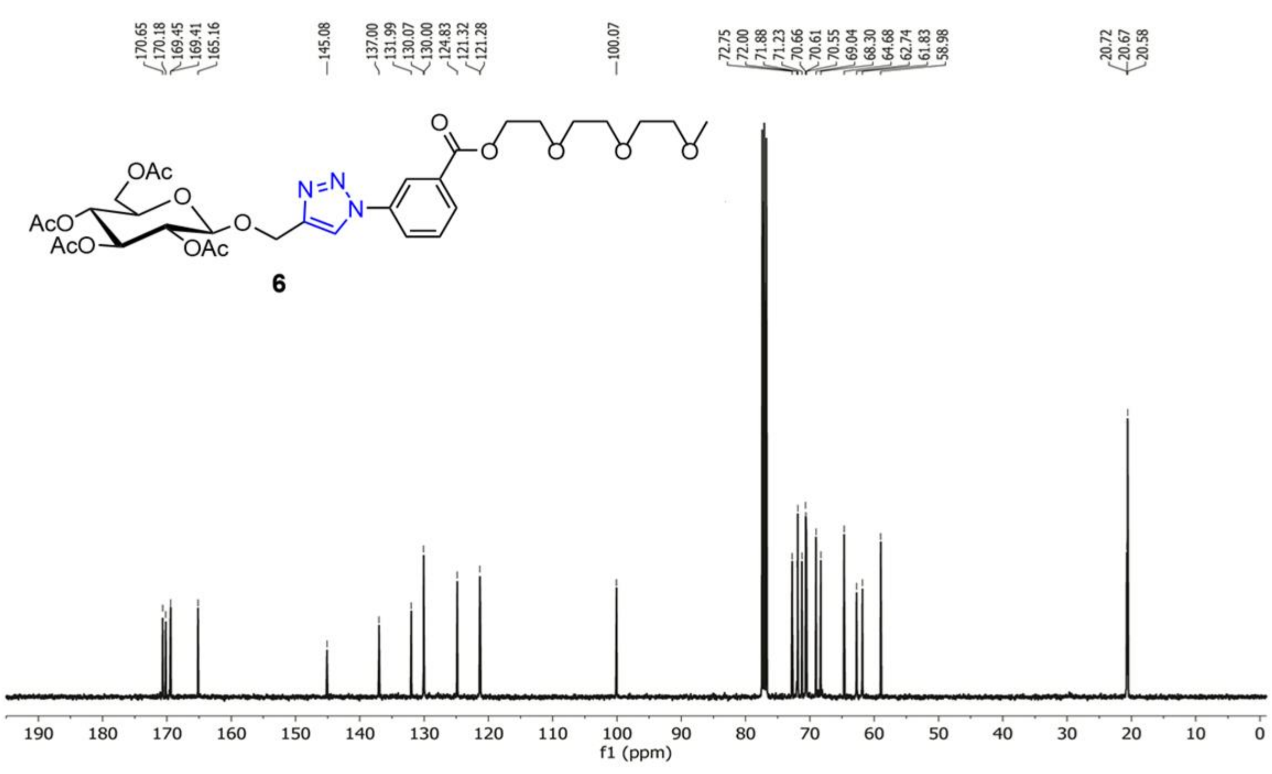

Figure 4. ^{13}C NMR spectrum of 1,2,3-triazole glucoside **6** ($CDCl_3$, 100 MHz).

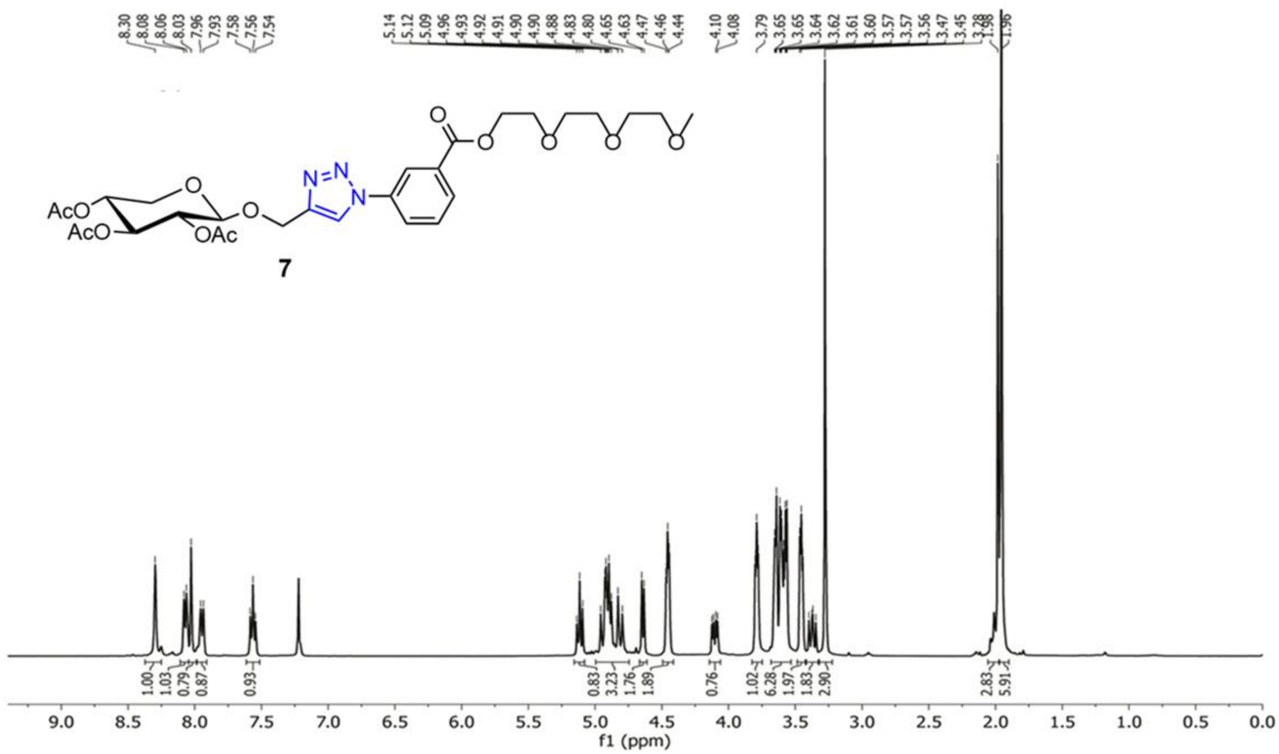

Figure 5. ^{1}H NMR spectrum of 1,2,3-triazole glucoside **7** ($CDCl_3$, 400 MHz).

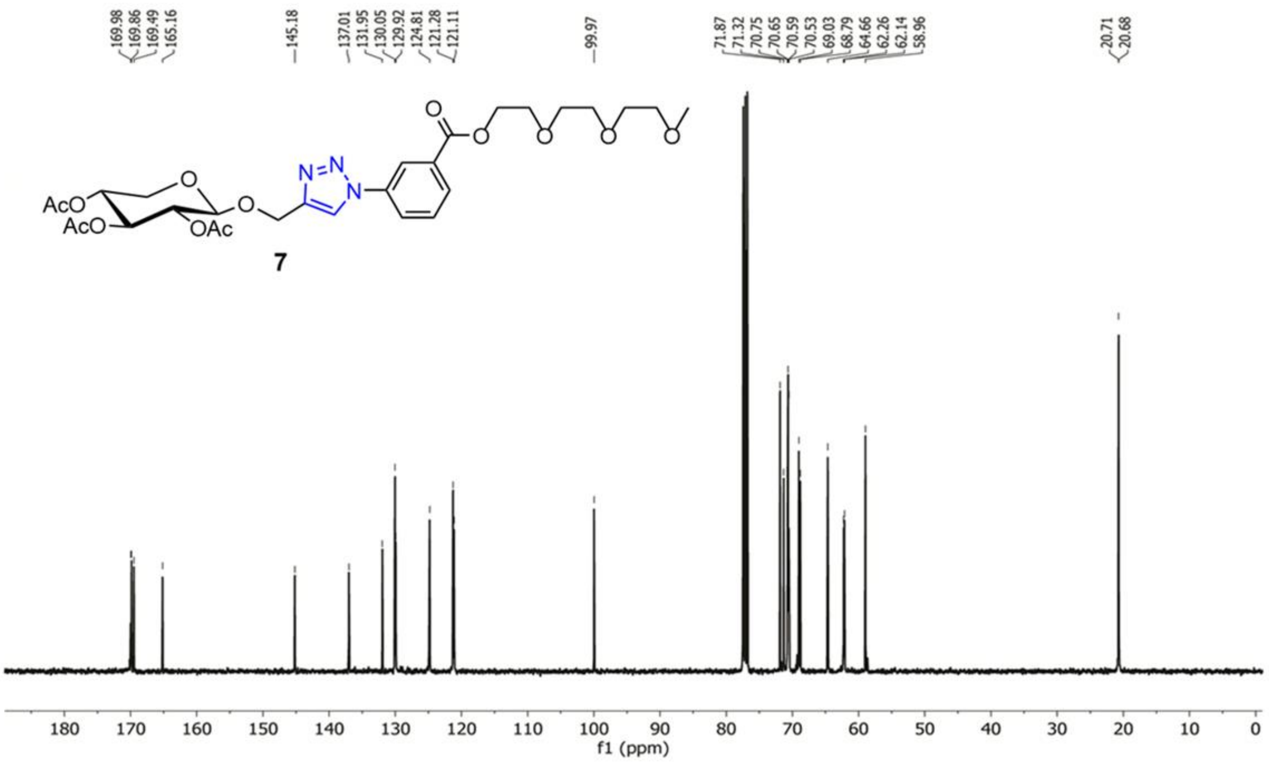

Figure 6. ^{13}C NMR spectrum of 1,2,3-triazole glucoside **7** ($CDCl_3$, 100 MHz).

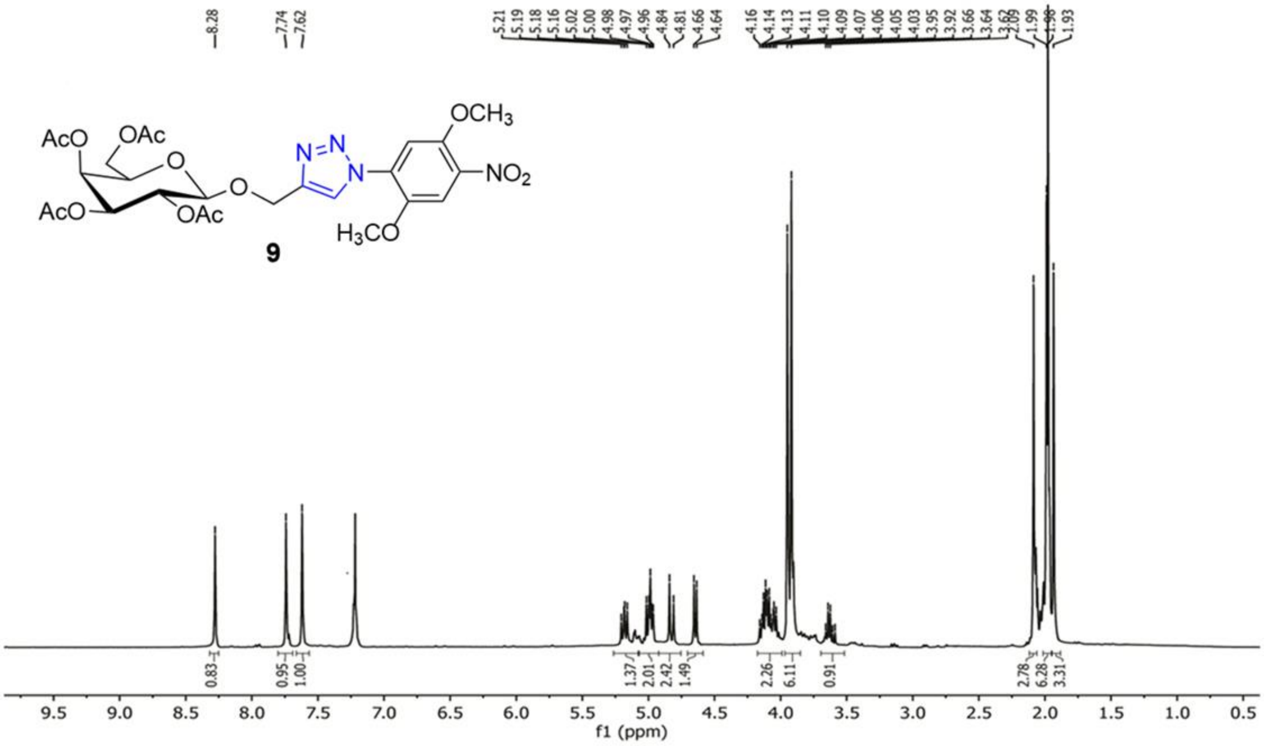

Figure 7. ^{1}H NMR spectrum of 1,2,3-triazole glucoside **9** ($CDCl_3$, 400 MHz).

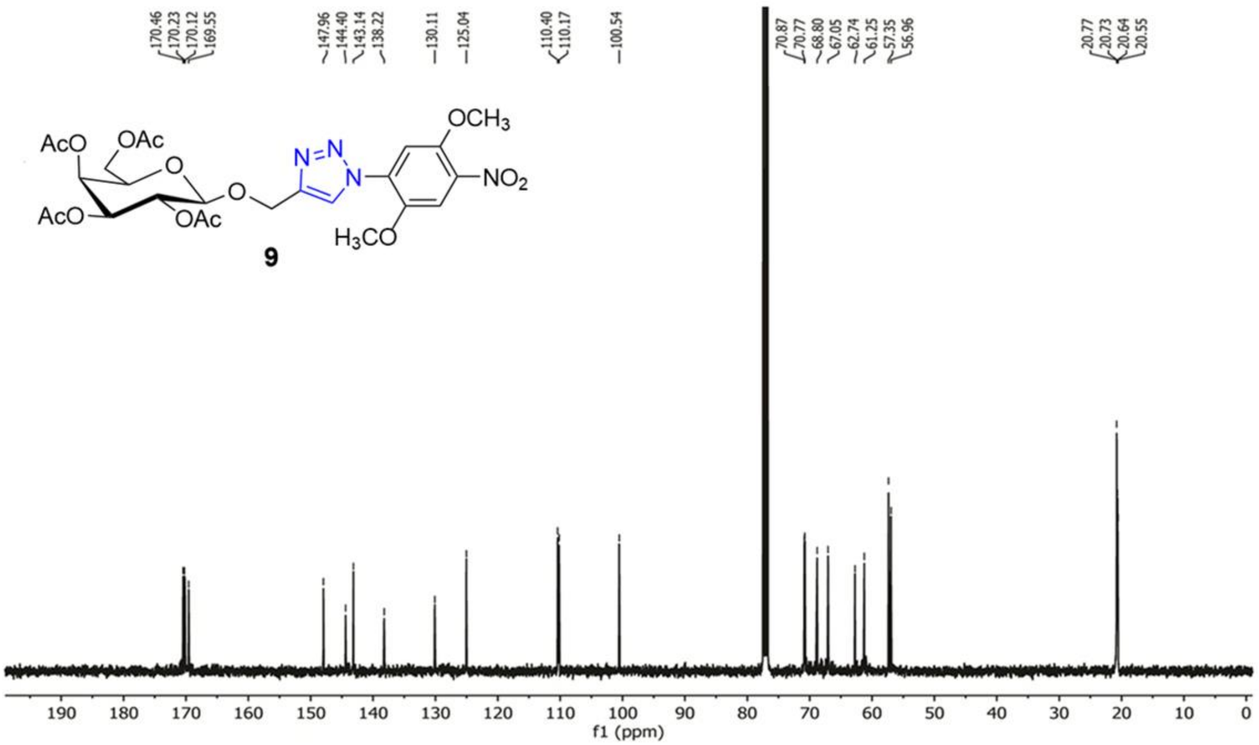

Figure 8. ^{13}C NMR spectrum of 1,2,3-triazole glucoside **9** ($CDCl_3$, 100 MHz).

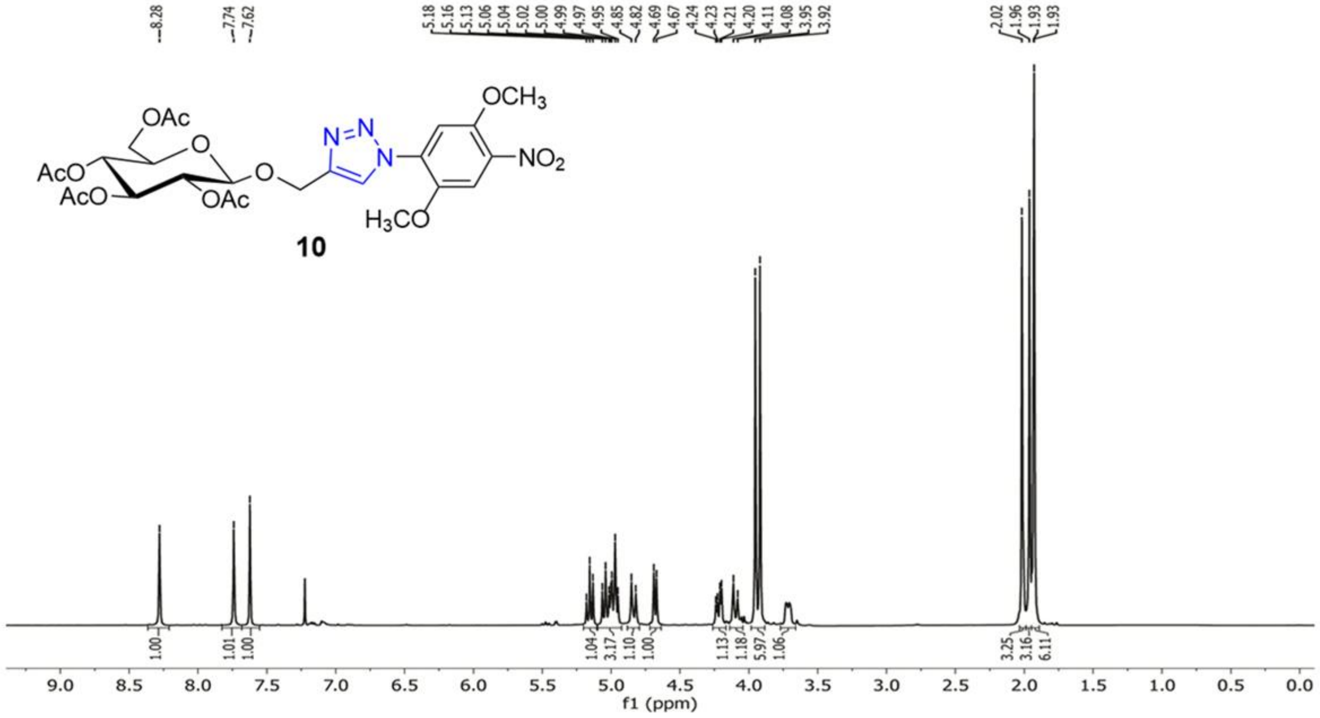

Figure 9. ^{1}H NMR spectrum of 1,2,3-triazole glucoside **10** ($CDCl_3$, 400 MHz).

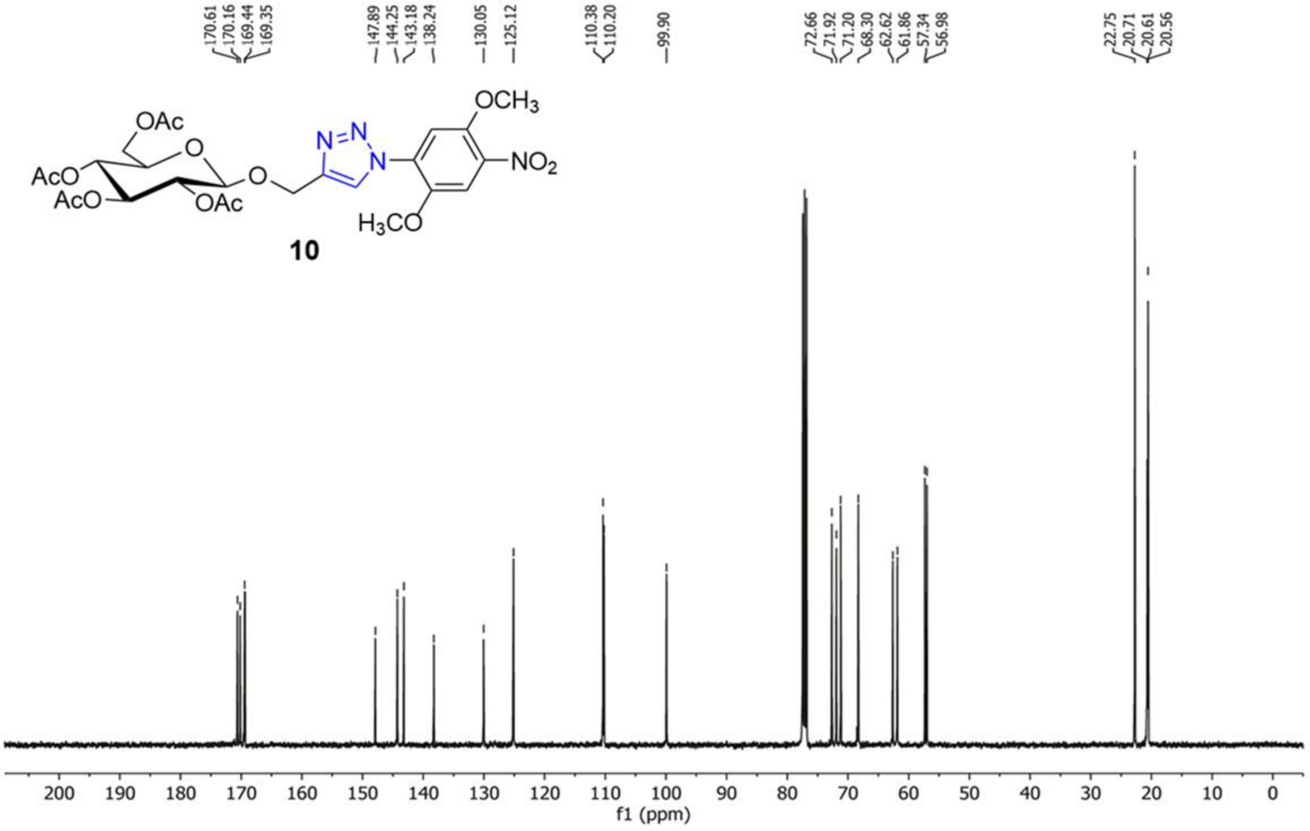

Figure 10. ^{13}C NMR spectrum of 1,2,3-triazole glucoside **10** ($CDCl_3$, 100 MHz).

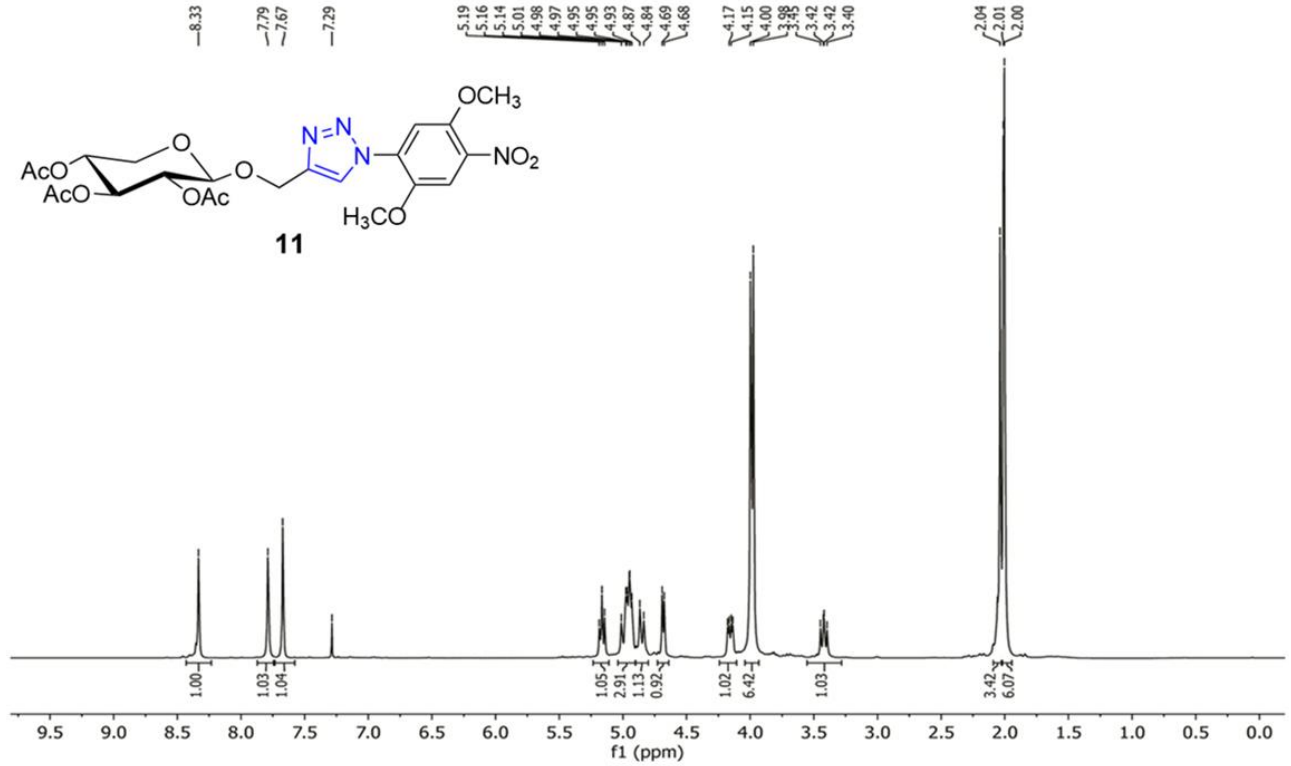

Figure 11. ^{1}H NMR spectrum of 1,2,3-triazole glucoside **11** ($CDCl_3$, 400 MHz).

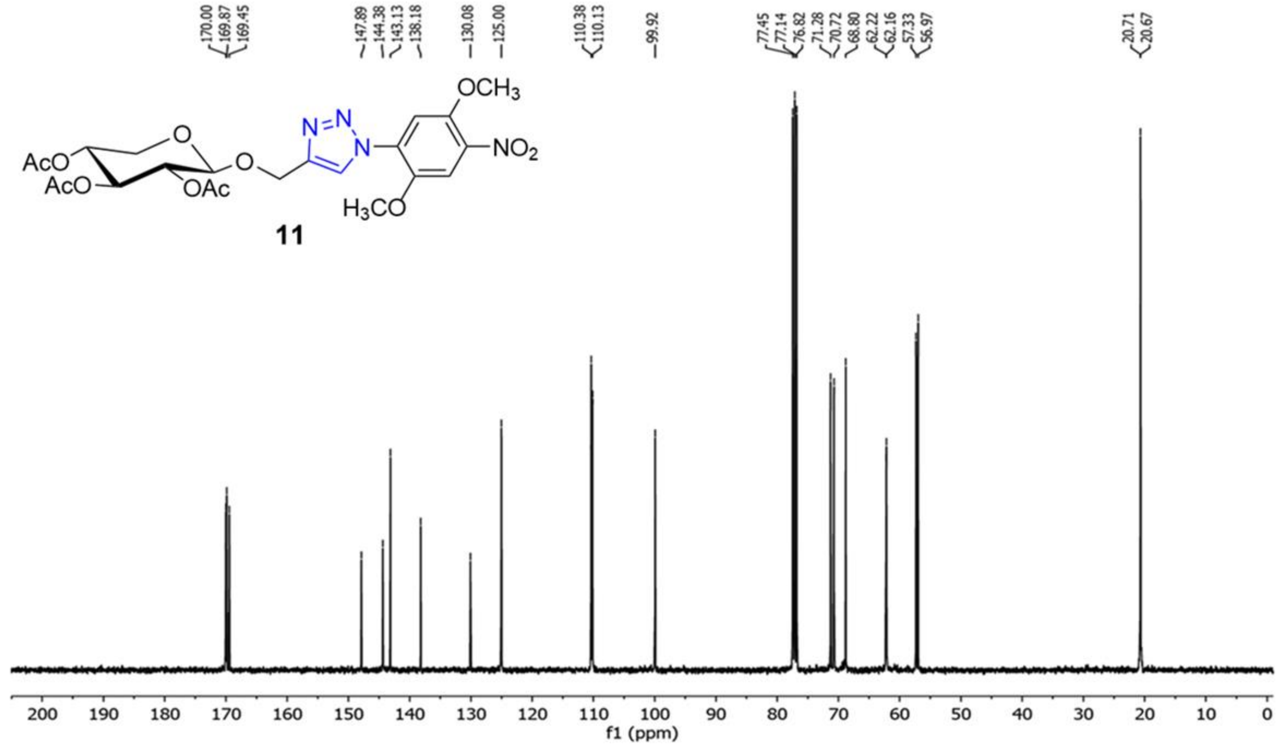

Figure 12. ^{13}C NMR spectrum of 1,2,3-triazole glucoside **11** ($CDCl_3$, 100 MHz).
